# Escape From X‐Chromosome Inactivation Enables Survival in a Male With an Unbalanced X;19 Translocation

**DOI:** 10.1111/nyas.70340

**Published:** 2026-07-11

**Authors:** Onur Emre Onat, Tayfun Ozcelik

**Affiliations:** ^1^ Beykoz Institute of Life Sciences and Biotechnology Bezmialem Vakıf University İstanbul Türkiye; ^2^ Department of Molecular Biology Bezmialem Vakıf University İstanbul Türkiye; ^3^ Department of Molecular Biology and Genetics Bilkent University Ankara Türkiye; ^4^ Neuroscience Program, Graduate School of Engineering and Science Bilkent University Ankara Türkiye; ^5^ Institute of Materials Science and Nanotechnology, National Nanotechnology Research Center Bilkent University Ankara Türkiye

**Keywords:** escape from X‐chromosome inactivation, skewed X‐chromosome inactivation, survival mechanisms, unbalanced X;autosome translocations

## Abstract

X;autosome translocations allow us to study how X‐chromosome inactivation (XCI) spreads into autosomal DNA in vivo. We re‐evaluated a male patient with an inherited unbalanced X;19 translocation to assess XCI‐associated silencing of the translocated chromosome 19 segment. High‐resolution SNP array analysis refined the rearrangement to a terminal 19p13.3 deletion of <0.21 Mb and a ∼95.4 Mb duplication spanning Xq11.1‐Xqter. XCI testing showed opposite patterns in mother and son: the balanced carrier mother preferentially inactivated the normal X chromosome, whereas the proband preferentially inactivated der(19), which carries the duplicated Xq containing the X‐inactivation center. To determine whether autosomal genes on der(19) were silenced, we performed allele‐specific expression assays across chromosome 19 loci. Several informative loci showed biallelic expression in the proband, suggesting incomplete silencing of the translocated autosomal segment. Informative and less clearly interpretable loci were interspersed without an obvious positional gradient, favoring gene‐specific variation in silencing susceptibility over a simple distance‐dependent model. These findings are consistent with incomplete and heterogeneous silencing across the autosomal segment and provide a plausible molecular explanation for survival with this otherwise severe unbalanced rearrangement.

## Graphical abstract

This study examines a rare inherited unbalanced X;19 translocation in a male proband and shows that silencing of the translocated chromosome 19 segment is incomplete. Allele‐specific expression analysis indicates that several informative chromosome 19 genes escape silencing, helping preserve essential autosomal dosage and likely contributing to survival in a chromosomal configuration usually incompatible with male development.

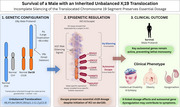

## 1 Introduction

X‐chromosome inactivation (XCI) is the central mechanism that balances X‐linked gene dosage between XX females and XY males. Early in embryonic development, one X chromosome in female cells is transcriptionally silenced through the action of the X‐inactivation center (XIC) and its key regulator, the long noncoding RNA *XIST*. Once expressed, *XIST* coats the chromosome in cis and recruits a series of chromatin‐modifying complexes that establish and maintain a heterochromatic state [1, 2]. The inactive X adopts characteristic epigenetic features, including CpG island hypermethylation, histone H4 hypoacetylation, loss of active chromatin marks, and delayed replication timing, that help lock in its silenced status across subsequent cell divisions [3].

Although the molecular framework of XCI on the X chromosome itself is now well established, much less is known about how the inactivation signal behaves when the XIC is repositioned onto an autosome. X;autosome translocations offer a rare natural experiment to study how chromatin context shapes the spread and stability of XCI. Early work, based largely on replication timing and cytogenetic assays, suggested that autosomal segments translocated onto the X can undergo only partial inactivation [4]. More recent studies using *XIST* RNA‐FISH and expression profiling have shown that the XCI signal often weakens or stops altogether as it crosses into autosomal DNA, implying that autosomal chromatin may resist complete silencing [5, 6, 7]. Despite these insights, the extent to which XCI spreads into autosomal material in vivo, and whether autosomal loci can maintain long‐term silencing once targeted by *XIST*, remain unresolved questions [8, 9].

Patterns of XCI in X;autosome translocations depend heavily on selective pressures. In balanced translocations, selection acts both against autosomal imbalance and against functional disomies of X‐linked genes, shaping the final XCI configuration [10]. Unbalanced X;autosome translocations, particularly in males, pose a far greater challenge. These rearrangements can produce functional monosomy or trisomy for large genomic regions and are usually incompatible with development [11]. Surviving males are exceptionally rare, and each case provides valuable insight into the relationship between gene dosage, XCI dynamics, and early developmental viability [12, 13, 14, 15, 16, 17, 18, 19, 20, 21]. To our knowledge, only two other inherited unbalanced X;autosome translocations in male patients have been documented (Table 1).

**TABLE 1:** Summary of reported cases of unbalanced X;autosome translocations in male patients identified in the literature.

Translocation	Sex	Karyotype	Rearrangement type	Clinical features	Inheritance	Ref.
X;6	Male	46, XY, t(X;6)(p22.1, p25)	Unbalanced	Multiple congenital anomalies	Inherited	[12]
X;9	Male	46, Y, der(X)t(X;9)(p22.32;p23)	Unbalanced	Brachytelephalangic type of chondrodysplasia punctata, mental retardation, and obesity	De novo	[13]
X;9	Male	46, XY, der(9)t(X;9)(p22.31;q34.3)	Unbalanced	Azoospermia	De novo	[14]
X;10	Male	46, XY, der(10)t(X;10)(q28;qter)	Unbalanced	Mental retardation, facial dysmorphic features, major axial hypotonia, severe feeding problems, and proneness to infections	De novo	[15]
X;10	Male	46, XY, der(10)t(X;10)(q28;p15.3)	Unbalanced	Prader–Willi‐like phenotype, feeding difficulties, hypotonia, and cryptorchidism	De novo	[16]
X;14	Male	47,Y,t(X;14)(q13;q14.32)	Unbalanced	A Klinefelter male	De novo	[17]
X;15	Male	46, XY, der(X)t(X;15)(p21.1;q11.2)	Unbalanced	Prader–Willi syndrome (PWS)‐like features, including hypotonia, hypogenitalism, hypopigmentation, and developmental delay	De novo	[18]
X;15	Male	46, XY, dic(X;15)(p11.1‐ q11.1; p13)	Unbalanced	Neurodevelopmental delay and phenotypic features	Inherited	[19]
X;19	Male	46, XY, der(19)t(X;19)(q11.1‐11.2;p13.3)	Unbalanced	Epilepsy, mental retardation, obesity, hypogonadism, Klinefelter‐like phenotype	Inherited	[20]
X;21	Male	46, XY, der(21)t(X;21)(q26;p11.2)	Unbalanced	Multiple congenital abnormalities, severe pre‐ and postnatal growth retardation, developmental delay, hypotonia, microcephaly, dysmorphic facial features, and cryptorchidism	Not determined	[21]

The proband described here was originally reported by Balci et al. [20] as a male with an inherited unbalanced X;19 translocation transmitted through a balanced maternal carrier. This rearrangement, consisting of an Xq duplication translocated onto 19p together with a small 19p deletion, offers a stringent opportunity to examine whether autosomal genes on an *XIST*‐containing derivative chromosome are silenced or able to escape.

In the present study, we extend the original report by applying high‐resolution SNV array mapping, quantitative XCI analysis, and allele‐specific expression assays. Our goals were to (i) refine the translocation breakpoints at a higher resolution, (ii) determine XCI patterns in the proband and his family members, and (iii) assess whether genes on the derivative chromosome 19 remain active or are subjected to XCI. Through this approach, we aimed to better understand how XCI spreads into, and is maintained on, autosomal DNA in a context that is typically incompatible with male survival.

## 2 Materials and Methods

### 2.1 Clinical Evaluation and Cytogenetic Analysis

The proband and family investigated here were previously described clinically and cytogenetically by Balci et al. [20]. The present study is a retrospective molecular follow‐up investigation based on archived samples from that original evaluation. Written informed consent and institutional ethics approval were obtained at the time of the initial clinical evaluation from the proband's legal guardians and participating family members. Archived peripheral blood‐derived DNA and RNA samples were obtained from the Hacettepe University DNA Bank, where the family's materials had been stored following the original clinical work‐up and made available for subsequent molecular analyses. No new samples were obtained and no further clinical or invasive procedures were carried out for this study. In this follow‐up investigation, we extend the original report by conducting high‐resolution SNV array mapping, XCI assays, and allele‐specific expression analyses using stored genomic DNA and complementary DNA (cDNA).

### 2.2 High‐Resolution SNV Array and Copy Number Analysis

Genomic DNA was isolated from 0.4 mL of peripheral blood using the NucleoSpin Blood kit (Macherey‐Nagel, Germany). DNA concentration and purity were checked by NanoDrop. Genotyping was carried out on the Affymetrix GeneChip Mapping 250K NspI platform following the manufacturer's instructions. Briefly, 250 ng of DNA from the proband (07‐966) and his mother (07‐967) was digested with *Nsp*I, adaptor‐ligated, PCR‐amplified, fragmented, labeled, and hybridized to the arrays. Processing was performed on the Affymetrix Fluidics Station 450 and scanned using the GeneChip Scanner 3000.

SNV calling and copy number variant (CNV) analysis were conducted with Affymetrix GCOS, GTYPE v4.0, CNAT v3.0.32, and CNAG v3.2.0.0 (Figure S1). Additional visualization was done with NEXUS Copy Number (v4.0) (Figure S2). Call rates were 87.5% for the proband and 95.5% for the mother. Reference data consisted of 175 healthy individuals, including 97 HapMap controls (89 CEU, 5 CHB/JPT, 3 YRI), 48 additional HapMap and non‐HapMap samples, 18 tumor–normal paired samples, and 12 X‐chromosome titration samples (trisomic and nontrisomic states). For CNAT/CNAG analyses, a subset of 93 healthy female controls from this cohort served as the reference group.

### 2.3 Gene Ontology Analysis

Genes located within the subtelomeric deleted interval (19p13.3‐19pter) (Table S1), the duplicated Xq11.1‐Xqter interval on the derivative chromosome, and the flanking translocation breakpoint region at Xq11.1‐11.2 (62.5−63.5 Mb) (Table S2) were retrieved using Ensembl BioMart. Gene ontology (GO) enrichment was assessed with the GOTree module of WebGestalt (Web‐based Gene Set Analysis Toolkit), with analyses focused on biological processes, including developmental pathways, regulatory functions, and responses to internal and external stimuli [22].

### 2.4 XCI Assay

XCI patterns were assessed using the androgen receptor (AR) methylation assay, which examines methylation at the polymorphic CAG repeat located at Xq12 and provides a localized readout of XCI skewing [23, 24]. Genomic DNA was isolated from 0.4 mL of peripheral blood, and 500 ng of genomic DNA was used for the AR methylation assay. DNA was digested with the methylation‐sensitive enzyme *Hpa*II, which selectively cuts unmethylated (active) alleles. PCR amplification of the AR locus was performed with primers 5′‐GTC CAA GAC CTA CCG AGG AG‐3′ and 5′‐CCA GGA CCA GGT AGC CTG TG‐3′. Digested and undigested PCR products were separated on 10% denaturing polyacrylamide gels (29:1 acrylamide:bisacrylamide) for 4 h at 15 W. Band intensities were quantified with MultiAnalyst v1.1. Corrected XCI ratios were calculated by dividing the upper/lower allele ratio of the digested sample by the ratio of the undigested sample to normalize for amplification differences. Ratios between 30:70 and 70:30 were interpreted as random XCI, whereas ratios greater than 95:5 were interpreted as extremely skewed XCI.

### 2.5 Selection of SNVs for Allelic Expression Analysis

Candidate SNVs were chosen based on two criteria: (i) informative minor allele frequencies (approx. 40%–50%) in European and Turkish populations, and (ii) confirmed expression in peripheral blood. Expression was verified using GTEx v9 whole‐blood TPM. Genomic coordinates for chromosome 19 genes were taken from Ensembl GRCh38. Distances from the 19p13.3 breakpoint were computed, and allelic expression results were plotted by genomic position to visualize whether escape patterns related to distance. Genes were further filtered to include SNVs with a restriction enzyme site suitable for distinguishing alleles. Final selection prioritized broad coverage across 19p and technical reliability for PCR and digestion. Candidate SNVs were selected on the basis of population allele frequency, peripheral blood expression, and technical suitability for restriction‐based allele discrimination; however, the proband's genotype at each analyzed SNV was not independently confirmed by separate genomic DNA genotyping. Alleles are reported throughout the manuscript according to dbSNP forward‐strand notation; restriction‐fragment sizes are listed separately for the corresponding digestion products. A complete list of SNVs, primer sequences, amplicon lengths, restriction enzymes, and expected fragment patterns is provided in Table S3. Primers were preferentially designed across exon–exon junctions to reduce the chance of genomic DNA contamination. Primer design used Primer3; restriction sites were verified with NEB Cutter (v2.0) and JustBio Cutter. Allele‐specific expression patterns were interpreted conservatively. A clear dual restriction‐fragment pattern in cDNA was considered evidence compatible with biallelic expression and escape from complete silencing. By contrast, a single visible restriction‐fragment pattern in the proband was not interpreted as definitive evidence of monoallelic expression or complete autosomal silencing, because the corresponding genomic genotype was not independently confirmed for each analyzed SNV. Such loci were, therefore, classified as noninformative or interpreted cautiously and were not used as evidence for the complete silencing of autosomal genes. Allelic expression patterns were evaluated on both agarose and polyacrylamide gels.

### 2.6 RNA Extraction, cDNA Synthesis, and RT‐PCR

Total RNA was extracted from 1.5 mL of peripheral blood collected in PAX tubes using the Qiagen RNA Blood Midi Kit, following the manufacturer's instructions. RNA quantity and purity were assessed using a NanoDrop spectrophotometer. cDNA was synthesized from 1 µg of total RNA with the RevertAid First Strand cDNA Synthesis Kit (MBI Fermentas). RT‐PCR was performed using gene‐specific primers flanking the selected SNVs. The resulting PCR products were digested with the appropriate restriction enzymes at 37°C for 4 h and separated on 2.5% agarose and 8% denaturing polyacrylamide gels. Digestion patterns were then examined to assess allele‐specific expression for each locus.

## 3 Results

### 3.1 Case Synopsis

**FIGURE 1:**
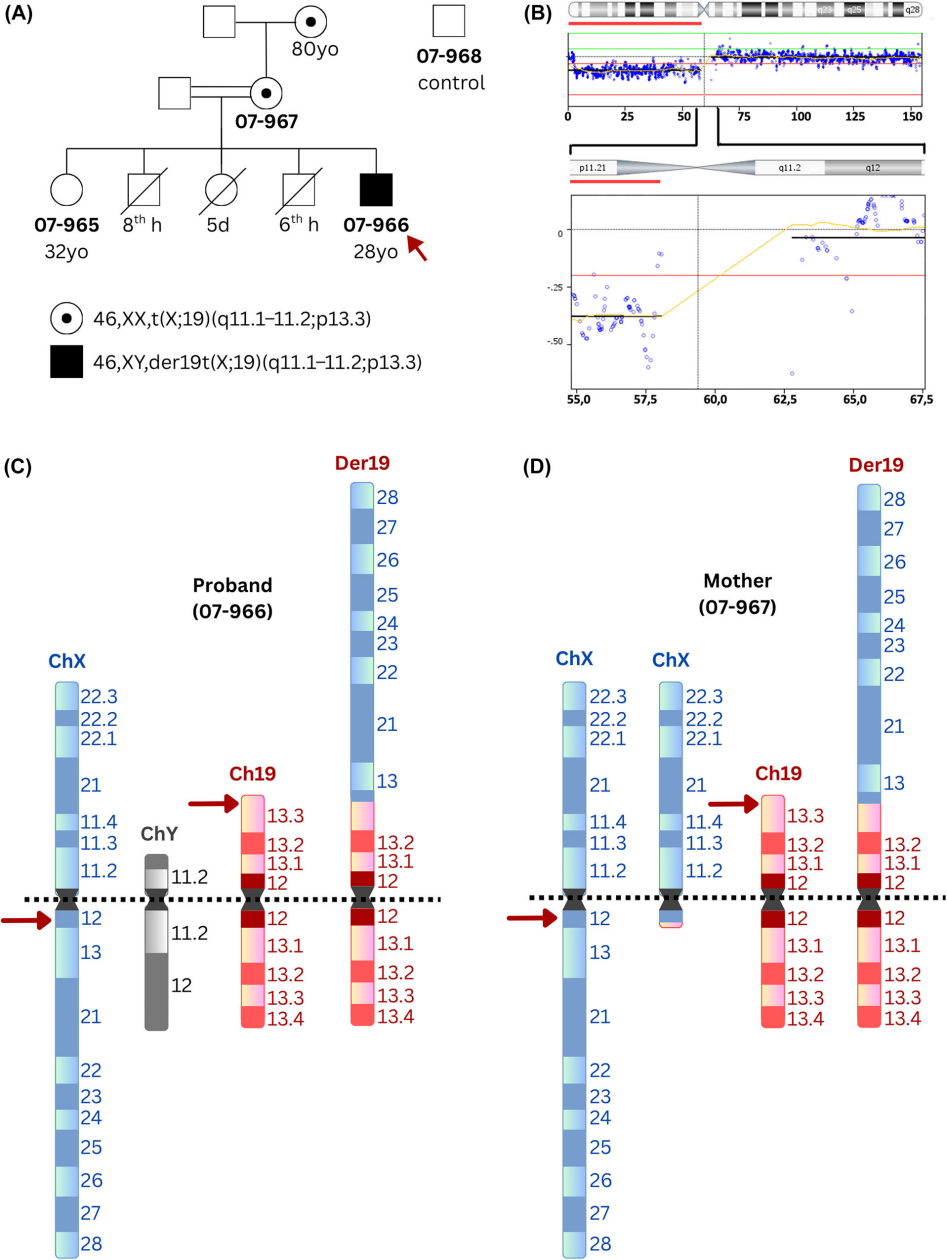
Clinical and cytogenetic overview of the family and rearrangement. (A) Pedigree of the family showing the proband with the unbalanced X;19 translocation (filled symbol). Individuals carrying the balanced translocation are indicated by dotted circles. (B) High‐density SNV array profile of the proband showing the large duplication along Xq. Log_2_ intensity ratios across the X chromosome confirm the presence of the duplicated segment. (C) Schematic representation of the derivative chromosome 19 in the proband (07‐966), carrying the duplicated Xq segment. (D) Schematic representation of the balanced X;19 translocation in the mother (07‐967). Red arrows indicate the approximate breakpoint positions.

The proband was a 26‐year‐old male with bilateral periventricular nodular heterotopia (bPNH), epilepsy, severe intellectual disability, obesity, and hypogonadism, whose clinical findings and initial cytogenetic and radiological features were previously detailed by Balci et al. [20]. Briefly, he was first referred for clinical evaluation because of seizures and episodes of self‐injurious behavior, which became apparent around age 6. Family accounts indicate that seizures began during infancy, followed by the development of severe intellectual disability, obesity, and hypogonadism. Brain magnetic resonance imaging (MRI) showed bPNH, a neuronal migration abnormality frequently linked to drug‐resistant epilepsy and neurodevelopmental impairment. The pedigree is shown in Figure 1A.

As originally described, fluorescence in situ hybridization (FISH) revealed an unbalanced X;19 translocation with the karyotype 46, XY, der(19)t(X;19)(q11.1–11.2;p13.3). His mother and maternal grandmother were identified as balanced translocation carriers (46, XX, t(X;19)(q11.1–11.2;p13.3)), confirming maternal transmission. Multicolor banding (MCB) provided additional structural resolution, showing that in the balanced carrier mother, der(19) contains the *XIST* locus (supported by two flanking probes) together with the subtelomeric region of 19p. However, the absence of major clinical manifestations in the carrier females is more likely explained by the balanced nature of the rearrangement together with preferential inactivation of the normal X chromosome, which preserves expression of the autosomal material on der(19) while avoiding dosage imbalance from the X‐derived segment on der(X), which lacks the XIC [20].

### 3.2 Refinement of Translocation Breakpoints

We initially focused on refining the translocation breakpoint regions in both the patient and his mother. The original study by Balci et al. [20] localized the breakpoints using subtelomeric FISH and confirmed their orientation by MCB. To achieve higher resolution, we applied Affymetrix 250K SNV array analysis, which confirmed the unbalanced translocation in the patient (Figure 1B). Consistent with earlier FISH and MCB data, the patient's karyotype was confirmed as 46, XY, der(19)t(X;19)(q11.1–11.2;p13.3) (schematically represented in Figure 1C) and the mother's karyotype as 46, XX, t(X;19)(q11.1–11.2;p13.3) (schematically represented in Figure 1D).

**FIGURE 2:**
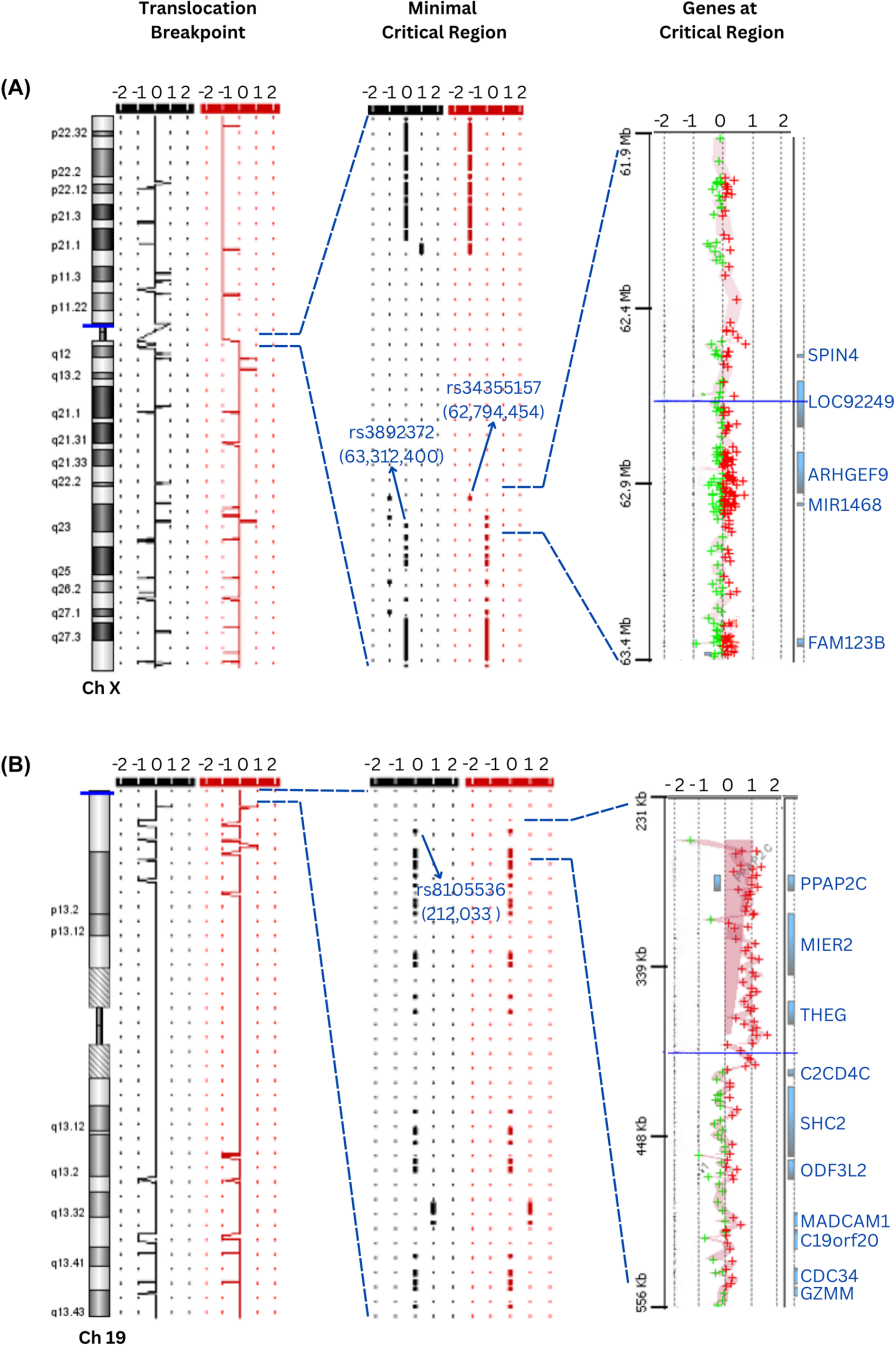
Refinement of the translocation breakpoints on chromosomes X and 19. (A) Affymetrix GeneChip SNV array profile of the X chromosome showing the breakpoint interval associated with the duplicated Xq segment. (B) Affymetrix GeneChip SNV array profile of chromosome 19 showing the terminal deleted interval at 19p13.3. In both panels, the proband's data are shown in red and the mother's data in black. Each point represents the copy‐number state of a single SNV determined by hidden Markov model (HMM) analysis. Breakpoint‐defining SNVs used to narrow the minimal critical regions are indicated by blue labels and arrows. Genes located within or adjacent to the breakpoint intervals are shown according to genomic order.

The original FISH/MCB work placed the chromosome 19 breakpoint within 19p13.3 (0–6.9 Mb) and the X‐chromosome breakpoint in Xq11.1–11.2 (59.5–65.1 Mb). SNV‐array analysis allowed us to narrow these intervals considerably. The translocation breakpoint resulted in a large duplication of X‐chromosomal material spanning approximately 95.4 Mb, beginning between rs34355157 (62.8 Mb) and rs3892372 (63.3 Mb) and extending to Xqter (Figure 2A). On chromosome 19, we detected a small terminal deletion of <0.21 Mb at 19p13.3–pter, proximal to marker rs8105536 (Figure 2B).

### 3.3 Gene Ontology of Affected Regions

**FIGURE 3:**
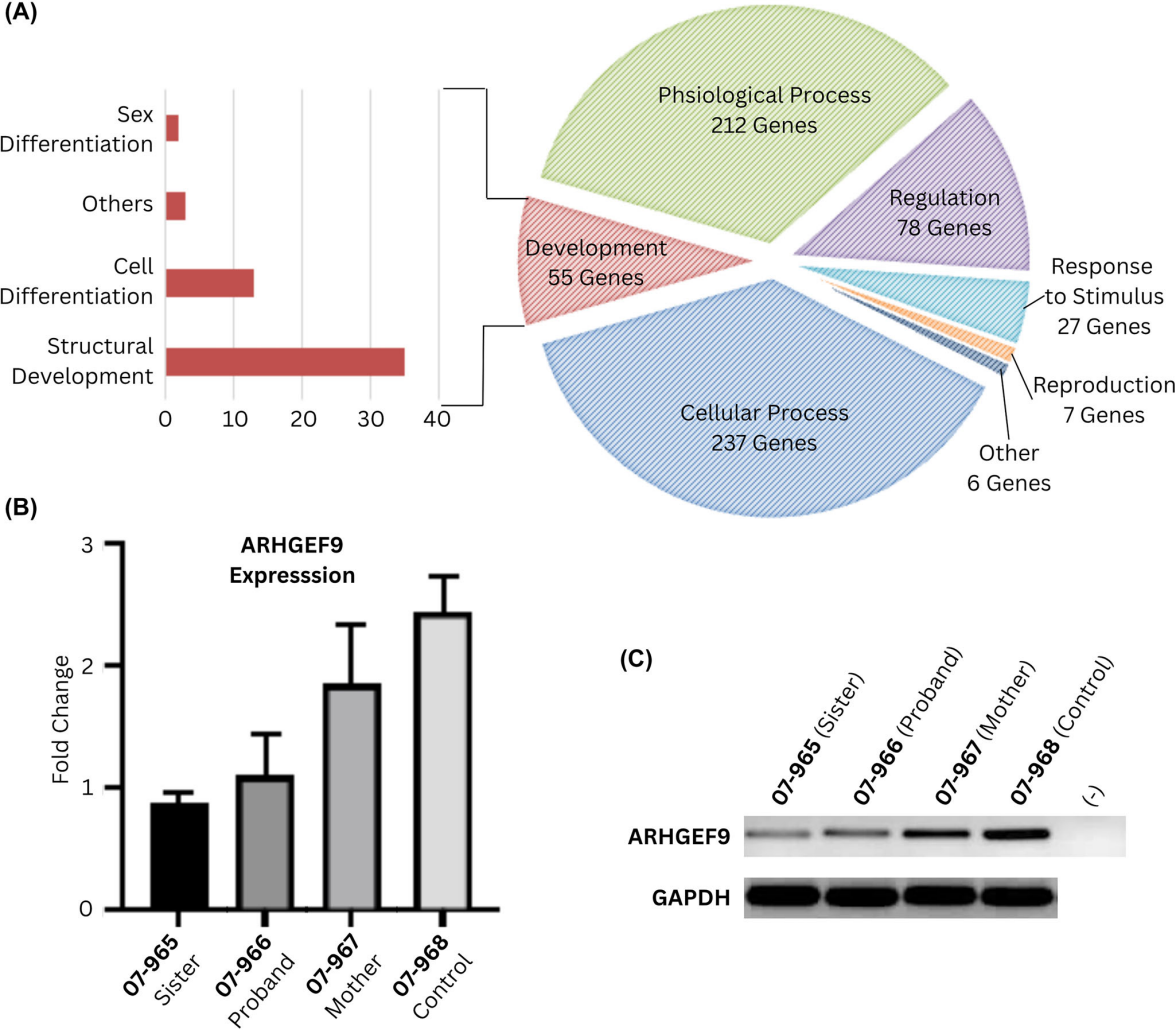
Gene ontology analysis of the duplicated Xq region and expression of *ARHGEF9*. (A) Gene ontology categories enriched among protein‐coding genes within the duplicated Xq11.1–Xqter region, generated using WebGestalt. (B) Relative *ARHGEF9* expression measured by quantitative RT‐PCR and normalized to *GAPDH* in the sister (07‐965), proband (07‐966), mother (07‐967), and a male control (07‐968). (C) Semi‐quantitative RT‐PCR of *ARHGEF9* in peripheral blood from the same individuals shown in panel B, with *GAPDH* shown as a control.

The subtelomeric deletion on 19p13.3 contained very few genes (Figure 2B and Table S1). In contrast, the duplicated Xq11.1‐qter segment contained 536 protein‐coding genes involved in a broad range of cellular (237 genes) and physiological (212 genes) processes, including development (55 genes), regulation (78 genes), reproduction (7 genes), and response to stimuli (27 genes). Thirty‐five of the 55 genes annotated under developmental processes were associated with structural development. A summary of enriched GO terms is presented in Figure 3A.

Because *ARHGEF9* (OMIM 300429), an X‐derived gene located near the X‐chromosomal breakpoint region, has been implicated in several neurodevelopmental disorders, we examined its expression as a representative candidate (Figure 2A and Table S2). *ARHGEF9* expression in the proband's blood was similar to that of a male control (Figure 3B), arguing against a major alteration of its leukocyte expression (Figure 3C). Given that *ARHGEF9* is subject to XCI, the lack of a clear increase in the proband is not unexpected. Expression appeared somewhat lower in the mother and sister; however, because the sister was not a carrier of the rearrangement, this pattern should not be interpreted as direct evidence of a translocation‐related regulatory effect. Instead, these modest differences may reflect interindividual variation, tissue‐specific regulation, or technical variability. Therefore, the breakpoint may not materially impair *ARHGEF9* expression in peripheral blood, although subtle or tissue‐specific effects, particularly in the brain, cannot be excluded. However, the small 19p deletion and genes directly disrupted by the breakpoint are unlikely to be the primary contributors to the clinical phenotype, whereas the Xq duplication encompasses numerous genes plausibly relevant to the neurological and endocrine features of the proband.

### 3.4 Skewed X‐Inactivation Patterns in Patient and Mother

**FIGURE 4:**
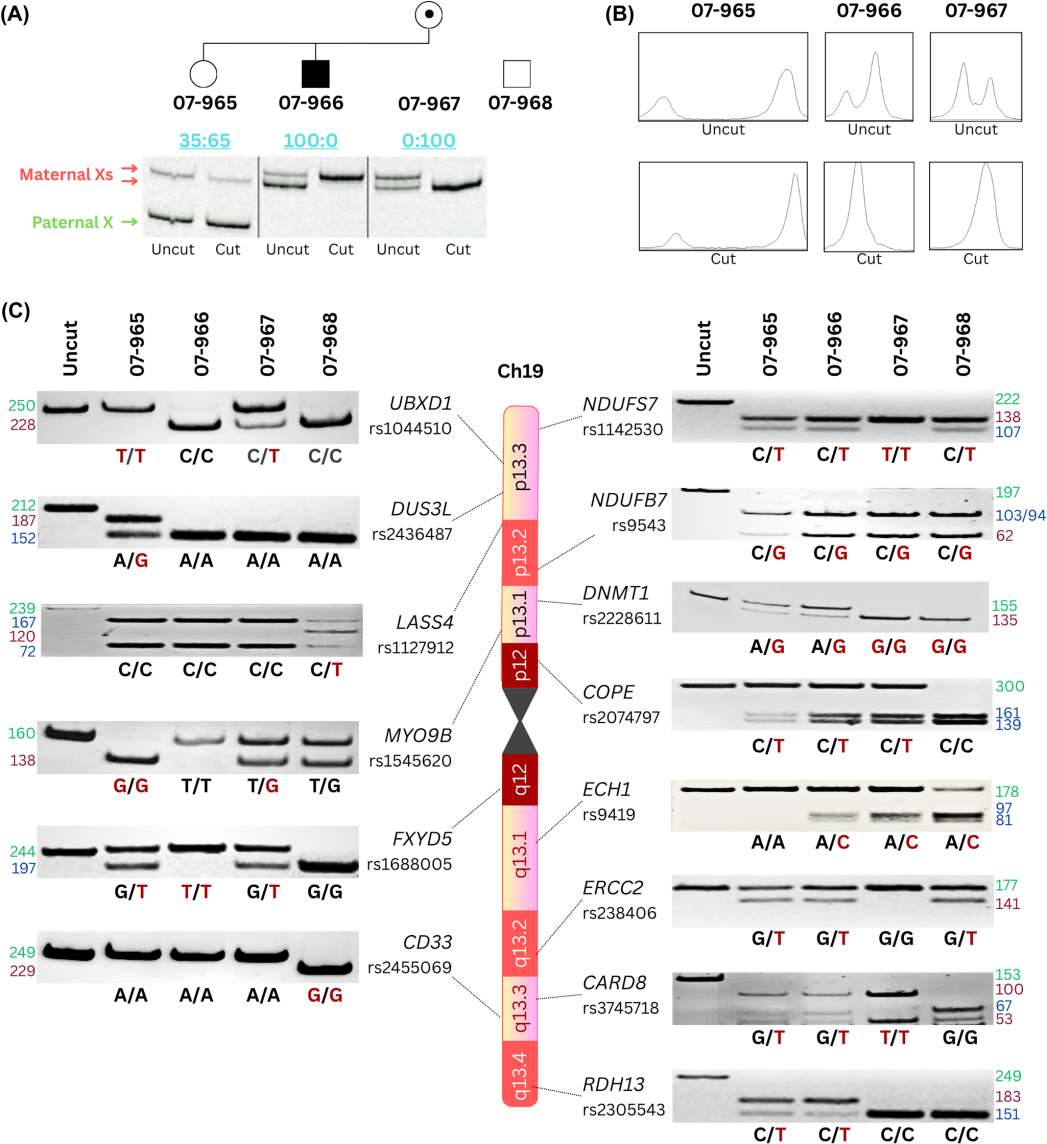
X‐chromosome inactivation patterns and allele‐specific expression analyses of selected chromosome 19 loci. (A) Methylation‐sensitive AR assay showing X‐chromosome inactivation (XCI) patterns in the sister (07‐965), the mother (07‐967), and the proband (07‐966). Maternal alleles are indicated by red arrows, and the paternal allele in the sister is indicated by a green arrow. XCI skewing ratios are shown in blue. (B) Densitometric traces corresponding to the AR methylation assay shown in panel A. (C) Allele‐specific cDNA restriction‐digestion patterns from selected chromosome 19 loci arranged by genomic position. Genotypes shown below each panel indicate the corresponding SNV state in the family. Loci that were homozygous in the proband were considered noninformative for evaluating allelic expression in him and are shown in the left panel. Fragment sizes corresponding to undigested and allele‐specific digestion products are indicated alongside each panel (font colors: uncut product, green; reference allele, blue; alternate allele, red). Semi‐quantitative densitometric measurements are summarized in Table S5. The clearest evidence supports escape from silencing at several informative loci, whereas evidence for robust silencing of chromosome 19 genes was limited in the present dataset.

To explore the epigenetic regulation of the rearranged chromosomes, we examined XCI using the methylation‐sensitive AR assay. The proband (07‐966) showed complete skewing toward inactivation of der(19), the derivative chromosome carrying the duplicated Xq containing the XIC, whereas his mother (07‐967) showed the opposite pattern, consistent with preferential inactivation of the normal X chromosome (Figure 4A). In the proband, the informative AR alleles allowed discrimination between the endogenous paternal X‐linked allele and the duplicated maternal Xq allele carried on der(19), enabling inference of preferential methylation/inactivation of the derivative chromosome. The sister (07‐965) exhibited a random pattern (35:65), consistent with that seen in healthy females (Figure 4B). She was not identified as a carrier of the X;19 translocation and is included here as an intrafamilial control for interpretation of *AR* alleles and allelic expression patterns.

The AR methylation assay was used here to determine the allelic pattern of XCI in informative individuals, specifically whether XCI was random or skewed. Although the assay interrogates methylation at the polymorphic AR locus, it should not be interpreted as a direct test of AR gene expression, locus‐specific escape, or the silencing status of individual X‐linked genes across the duplicated Xq segment. In the present study, the purpose was to establish the direction and degree of XCI skewing in the family. Therefore, differences observed at other loci should be interpreted as locus‐specific transcriptional outcomes rather than as regional differences in XCI skewing itself. Nonetheless, the opposite skewing patterns observed in mother and son are consistent with known selective pressures in X;autosome rearrangements [7]. In the mother, preferential inactivation of the normal X chromosome would preserve expression of the autosomal material translocated onto der(19), while also avoiding unsilenced dosage excess from the X‐derived segment on der(X), which lacks the XIC. In the proband, inactivation of der(19) would help reduce excessive dosage from the duplicated Xq material. These contrasting patterns support the idea that different selective requirements act in balanced translocation carriers versus individuals with unbalanced X;autosome translocations.

### 3.5 Evidence for Incomplete Silencing of the Translocated Chromosome 19 Segment

Chromosome 19 is the most gene‐dense human chromosome, enriched for CpG islands, GC‐rich regions, and repetitive elements [25]. It contains 1461 protein‐coding genes and 312 pseudogenes, including many with essential cellular functions. Complete inactivation of the autosomal material on der(19) would, therefore, be incompatible with viability, raising the question of whether key chromosome 19 genes in this region remained active despite the chromosome's association with an inactive X.

We examined allele‐specific expression for 14 chromosome 19 genes using SNV‐based RT‐PCR followed by restriction enzyme digestion (Table 2 and Figure 4C). Because the proband's genotype at each analyzed SNV was not independently confirmed by separate genomic DNA genotyping, the assay was interpreted conservatively. The strongest biological inferences were, therefore, drawn from loci showing clear allele‐resolved cDNA digestion patterns that could be compared across family members and were most consistent with biallelic expression. These loci provided the clearest evidence for escape from silencing in the proband. By contrast, loci classified as monoallelic or noninformative were interpreted more cautiously, because without independent genotype confirmation, true monoallelic expression cannot always be distinguished from lack of informativeness. Accordingly, such loci were not used as equally strong evidence for XCI‐mediated silencing.

**TABLE 2:** Selected SNVs and corresponding genes chosen for allelic expression analysis based on specific characteristics.

Gene	Bld Exp	Chr	SNV ID	Syn	Shift	Allele	Het	Position
*NDUFS7*	260	19p13.3	rs1142530	No	P/L	(C/T)	0.495	1339538
*UBXD1*	50	19p13.3	rs1044510	Yes	—	(C/T)	0.442	4408650
*DUS3L*	100	19p13.3	rs2436487	No	R/G	(A/G)	0.473	5740565
*LASS4*	75	19p13.2	rs1127912	Yes	—	(C/T)	0.465	8227937
*DNMT1*	151	19p13.2	rs2228611	Yes	—	(A/G)	0.495	10128077
*MAST1*	42	19p13.2	rs2290688	Yes	—	(A/G)	0.497	12819697
*NDUFB7*	260	19p13.1	rs9543	Yes	—	(C/G)	0.491	14543804
*MYO9B*	117	19p13.1	rs1545620	No	S/A	(T/G)	0.416	17164774
*COPE*	227	19p13.1	rs2074797	Yes	—	(C/T)	0.440	18872244
*FXYD5*	126	19q12	rs1688005	No	S/A	(G/T)	0.500	40340205
*ECH1*	227	19q13.1	rs9419	No	E/A	(A/C)	0.482	44013927
*ERCC2*	109	19q13.3	rs238406	Yes	—	(G/T)	0.461	50560149
*CARD8*	134	19q13.3	rs3745718	Yes	—	(G/T)	0.453	53406965
*CD33*	50	19q13.3	rs2455069	No	R/G	(A/G)	0.457	56420453
*RDH13*	84	19q13.4	rs2305543	Yes	—	(C/T)	0.425	60251527

Abbreviations: Bld Exp, blood expression (GTEx v9); Chr, chromosome position; Het, heterozygosity; SNV, single nucleotide variant; Syn, synonymous status.

Loci that were homozygous in the proband were retained to document assay design and family segregation patterns, but were not considered informative for evaluating allelic expression in him (*UBXD1*, *DUS3L*, *LASS4*, *MYO9B*, *FXYD5*, and *CD33*). In addition, some loci showed variable band quality or closely spaced restriction products that limited confidence in quantitative interpretation. Among the informative loci, the strongest evidence supported predominant biallelic expression for a subset of genes, most clearly including *COPE* and *ERCC2. NDUFS7, NDUFB7, DNMT1*, and *RDH13* also showed patterns more consistent with escape than with complete silencing, although the band intensities were less cleanly resolved in the composite gel image. Additional loci, including *ECH1* and *CARD8*, were broadly compatible with biallelic expression or incomplete silencing, but the signal quality and lane‐to‐lane variability were not sufficient for strong quantitative inference (Tables S4 and S5). Overall, the allele‐specific expression data support substantial escape from silencing among several informative chromosome 19 genes in the proband, rather than broad or uniform inactivation of the translocated autosomal segment.

When the tested loci were mapped by distance from the 19p13.3 breakpoint, loci showing stronger evidence of escape and loci that remained less clearly interpretable were interspersed without an obvious positional gradient (Figure 4C). Although the number of loci was limited, this pattern is more consistent with gene‐specific variation in silencing susceptibility than with a simple distance‐dependent decay model.

Collectively, these findings indicate that the autosomal portion of der(19) is not uniformly silenced. Rather, the data support escape from silencing at several informative chromosome 19 loci, with locus‐to‐locus variability in the strength and interpretability of the allelic expression patterns. This partial preservation of autosomal gene expression likely contributed to survival in a chromosomal configuration that would otherwise be expected to be developmentally unfavorable.

## 4 Discussion

Building on the original clinical and cytogenetic description of this family [20], we set out to examine how XCI behaves when a large Xq fragment is translocated onto chromosome 19. The main conclusion from our work is that allele‐specific expression patterns at several informative chromosome 19 loci are consistent with escape from complete XCI‐associated silencing in the proband. Their continued expression likely provided enough dosage for survival, despite a chromosomal configuration that is typically incompatible with early development.

In total, we examined 14 chromosome 19 genes for allele‐specific expression, although not all loci were equally informative for interpretation in the proband. The clearest evidence for escape from silencing was observed at several informative loci, particularly *COPE* and *ERCC2*, with additional but less robust support at *NDUFS7*, *NDUFB7*, *DNMT1*, and *RDH13*. These findings support the view that silencing of the autosomal segment linked to der(19) is incomplete and gene dependent. However, we did not directly assess *XIST* RNA localization in patient cells, and, therefore, cannot conclude that the translocated chromosome 19 region was physically coated by *XIST* RNA in the proband. A more cautious interpretation is that XCI was initiated on der(19), but that the spread and/or maintenance of silencing into autosomal chromatin was incomplete. Importantly, this locus‐specific variation should not be confused with regional variation in XCI along the X chromosome itself; rather, the skewing event is chromosome‐wide, whereas transcriptional outcomes at individual loci may vary depending on local genomic context. Whether this pattern reflects incomplete initiation of silencing, instability of maintenance over developmental time, or a combination of both cannot be resolved from our data. Overall, the results indicate that XCI does not extend uniformly across the derivative chromosome and that several chromosome 19 genes remain transcriptionally active despite the inactivation context of der(19). These findings are consistent with prior evidence that autosomal chromatin may be a suboptimal substrate for stable *XIST*‐mediated silencing, limiting the extent and persistence of XCI spread into autosomal DNA [26, 27].

This interpretation fits well with earlier studies of unbalanced X;autosome translocations, where *XIST* RNA, heterochromatin marks, and replication timing changes often weaken as they enter autosomal DNA [8, 9, 10]. Several factors have been proposed to underlie this attenuation, such as differences in chromatin organization [28], replication timing domains [29], and the relative scarcity of the repeat‐rich elements, particularly LINE‐1 sequences, that are thought to facilitate the spread of XCI along the X chromosome [30, 31]. Autosomes generally lack the dense repeat architecture and perinuclear positioning signals characteristic of the inactive X, and these features may collectively act as natural boundaries that limit the extent of *XIST*‐mediated silencing. It is, therefore, not surprising that the autosomal segment on der(19) proved resistant to complete inactivation.

The XCI patterns observed in the family are also consistent with known selective pressures in X;autosome rearrangements. The proband's mother, who carries the balanced translocation, showed preferential inactivation of the normal X chromosome, which is the pattern expected in balanced X;autosome translocations. This preserves expression of the autosomal material translocated onto der(19) and avoids the potentially deleterious consequences of leaving active the X‐derived segment on der(X) that lacks the XIC. Consistent with the original clinical/cytogenetic report, der(19) carries the *XIST* locus structurally; however, that observation should not be taken as direct evidence of *XIST* RNA coating across the autosomal segment in the present study [20]. The proband inactivated der(19), the derivative chromosome carrying the duplicated Xq material together with the *XIST*‐containing region. This pattern would help reduce X‐linked dosage excess, although complete silencing of the autosomal component would not be compatible with normal cellular function, making escape from silencing essential for survival. This scenario illustrates how partial or heterogeneous spreading of XCI can accommodate otherwise incompatible imbalances [32]. Whether inactive‐X heterochromatin physically extends into the 19p segment remains to be determined; approaches such as *XIST* RNA‐FISH or H3K27me3 immuno‐FISH would directly answer this question and represent an important next step. A plausible model is that during early embryogenesis, the embryo experienced selective pressure favoring cell populations in which XCI did not fully extend into the autosomal region. Cells in which essential 19p genes were silenced would have been lost early. This kind of developmental selection has been observed in other X;autosome rearrangements and suggests that XCI is not a fixed, all‐or‐nothing process, but a dynamic state influenced by chromatin context and developmental timing [32].

Among the genes showing escape from silencing, *DNMT1* is noteworthy because of its essential role in maintaining DNA methylation and epigenetic stability [33]. In the context of this rearrangement, continued *DNMT1* expression is more likely to be protective than deleterious, as escape from silencing would preserve expression of a dosage‐sensitive autosomal gene that would not be expected to tolerate complete inactivation. Thus, the observation of biallelic *DNMT1* expression is most plausibly interpreted as evidence that essential chromosome 19 gene dosage is at least partially preserved in the proband. Because *DNMT1* lies within an Alu‐rich region, it remains possible that local sequence context contributes to its escape behavior. Although local chromatin or sequence context may plausibly influence locus‐specific escape behavior, these features were not directly examined in the present study.

Despite his survival, the proband displays a severe clinical phenotype, including epilepsy, intellectual disability, obesity, hypogonadism, and bPNH. We considered several mechanisms to explain these findings. Disruption of genes at the breakpoint seems less likely to fully explain the phenotype, as the deleted 19p region is gene‐poor, and expression of the nearby X‐derived gene *ARHGEF9*, which has been associated with neurodevelopmental disorders [34, 35, 36]. *ARHGEF9* expression did not appear markedly altered in the proband's blood. Although *ARHGEF9* expression appeared somewhat lower in the mother and sister, the sister was not a carrier of the rearrangement; therefore, this observation cannot be directly attributed to the X;19 translocation itself and should be interpreted cautiously.

One possible contributor to the phenotype is residual dosage imbalance from the duplicated Xq segment. Because the duplicated Xq contains the XIC, preferential inactivation of der(19) in the proband would be expected to reduce X‐linked dosage excess. However, the present study did not directly assess allele‐specific expression of X‐linked genes within the duplicated Xq interval. Therefore, we cannot define which X‐linked genes, if any, escape or incompletely undergo XCI in this individual. The duplicated Xq region may still contribute to the phenotype through dosage‐sensitive genes that normally escape XCI or are incompletely silenced, but this remains inferential.

A second, mechanistically distinct consideration is the autosomal chromosome 19 material carried on der(19). Our allele‐specific expression data indicate that several informative chromosome 19 loci remain expressed, supporting incomplete silencing of the autosomal segment and providing a plausible explanation for survival. At the same time, the present assay is better suited to identifying escape from silencing than to proving complete silencing at individual autosomal loci. Therefore, variable dysregulation of chromosome 19 genes, including possible partial silencing at loci not captured by our assays, may also have contributed to the clinical phenotype.

Importantly, this case differs from previously reported distal Xq duplication cases that lack the XIC and, therefore, do not undergo XCI across the duplicated Xq segment. Those cases are useful for phenotypic comparison at a descriptive level, but they are not mechanistically equivalent to the present rearrangement, in which the duplicated Xq contains the XIC and the derivative chromosome also includes autosomal material. Accordingly, the phenotype cannot be attributed solely to the Xq duplication, and is more plausibly explained by the combined consequences of residual X‐linked dosage effects and dysregulation of the translocated autosomal chromosome 19 segment [32].

More broadly, our findings highlight how epigenetic mechanisms can buffer chromosomal imbalances during early development. The escape of critical autosomal genes from XCI demonstrates that cells can modulate silencing outcomes to preserve viability, even when presented with a potentially lethal rearrangement. These principles have implications beyond rare structural rearrangements and may also help explain patterns of resilience seen in other contexts, such as mosaicism, cancer, and sex chromosome aneuploidies [32].

There are, however, limitations. Our assays were performed in peripheral blood, and only a subset of chromosome 19 genes could be tested. Mosaicism cannot be excluded in tissues not sampled. We used the AR methylation assay to assess skewed versus random XCI patterns between informative alleles. This assay does not directly determine AR gene expression, locus‐specific escape, or the silencing status of individual X‐linked genes or the duplicated Xq segment as a whole. We did not assess allele‐specific expression of X‐linked genes within the duplicated Xq region; doing so would help define the boundaries of XCI and escape more precisely. In addition, because the proband's genotype at the analyzed SNVs was not independently confirmed by separate genomic DNA genotyping, loci showing apparent monoallelic or noninformative patterns could not be interpreted with the same confidence as loci showing clear biallelic expression. Accordingly, the present data are better suited to identifying escape from silencing than to proving locus‐specific autosomal silencing. Thus, although escape from silencing likely preserved essential autosomal gene dosage and contributed to survival, the phenotype itself may still reflect partial silencing of other chromosome 19 genes not fully captured in the present analysis.

In conclusion, our study illustrates the limits of *XIST*‐mediated silencing on autosomal material and is consistent with the possibility that local genomic context contributes to locus‐specific XCI outcomes. Escape of key chromosome 19 genes was almost certainly crucial for survival in this individual with an otherwise lethal unbalanced X;19 translocation. X;autosome translocations continue to provide unique insight into the interplay between chromatin regulation, dosage compensation, and developmental adaptation, and they remain valuable natural models for dissecting fundamental aspects of XCI biology.

## Author Contributions

O.E.O. conceived the study, designed the project, carried out the experiments and analyses, and drafted the initial manuscript. T.O. contributed to the study concept, project organization, and critical revision of the manuscript. All authors reviewed and approved the final version of the manuscript.

## Funding

No external funding was received for this study. The authors have no financial disclosures to report.

## Conflicts of Interest

The authors declare no conflicts of interest.

## Ethics Statement

Written informed consent and institutional ethics approval for the family's initial clinical assessment were obtained at the time of the original evaluation, as previously reported by Balci et al. (2007). The present study used only archived peripheral blood–derived DNA and RNA samples obtained from the Hacettepe University DNA Bank, where the family's materials had been stored and made available for subsequent molecular analyses. No new samples or interventions were undertaken for the present work. All procedures were conducted in accordance with institutional standards and the Declaration of Helsinki.

## Websites and Data Download Links

• HapMap controls, n97 (89 CEU, 5 CHB+JPT, 3 YRI): https://ftp.ncbi.nlm.nih.gov/hapmap/

• HapMap samples, n48 (5 CEPH trios, 5 YRI trios, 3 non‐HapMap trios and 9 unrelated Asian): https://www.thermofisher.com/in/en/home/technical-resources/technical-reference-library/microarray-analysis-support-center.html

• CNV Reference Data, n34 (18 tumor‐normal paired and 12 X‐chromosome titration samples; 4 aneuploid chromosome): https://www.thermofisher.com/in/en/home/life-science/microarray-analysis/microarray-data-analysis/microarray-analysis-sample-data.html

• Web‐based Gene set analysis toolkit (WebGestalt), GOTree tool: https://www.webgestalt.org/

• Ensembl BioMart database: www.ensembl.org/biomart/martview/

• NEB Cutter: https://nc3.neb.com/NEBcutter2/

• JustBio Cutter tool: http://www.justbio.com/cutter/index.php

• Primer3 tool: https://primer3.ut.ee

## Supporting information




**Table S1**. Genes located within the deleted subtelomeric interval at 19p13.3 (0–500 kb), extracted from Ensembl BioMart (version 0.7).
**Table S2**. Genes flanking the translocation breakpoint on Xq11.1–11.2 (62.5–63.5 Mb), extracted from Ensembl BioMart (version 0.7).
**Table S3**. Primer sets and restriction enzymes used for allele‐specific expression assays.
**Table S4**. Summary of semi‐quantitative interpretation of the allele‐specific expression assays shown in Figure 4.
**Table S5**. Semi‐quantitative densitometric summary of informative allele‐specific expression assays shown in Figure 4.
**Figure S1**. CNAG‐based visualization of single nucleotide variant (SNV) array data for the X chromosomes of the unbalanced X;19 translocation patient and the balanced carrier mother.
**Figure S2**. Nexus‐based visualization of Affymetrix SNV array data in the proband (07‐966).

## Data Availability

All data supporting the findings of this study are included in the article and its Supplementary Materials. Additional raw data can be provided by the authors upon reasonable request and without undue restrictions.
