# Supplementary material for: Escape From X‐Chromosome Inactivation Enables Survival in a Male With an Unbalanced X;19 Translocation
Source: Ann N Y Acad Sci. 2026 Jul 11;1561(1):e70340. doi: 10.1111/nyas.70340 (PMC13355919; doi:10.1111/nyas.70340)
Supplement: Supplementary file 1 — Table S1. Genes located within the deleted subtelomeric interval at 19p13.3 (0–500 kb), extracted from Ensembl BioMart (version 0.7). Table S2. Genes flanking the translocation breakpoint on Xq11.1–11.2 (62.5–63.5 Mb), extracted from Ensembl BioMart (version 0.7). Table S3. Primer sets and restriction enzymes used for allele‐specific expression assays. Table S4. Summary of semi‐quantitative interpretation of the allele‐specific expression assays shown in Figure 4. Table S5. Semi‐quantitative densitometric summary of informative allele‐specific expression assays shown in Figure 4. Figure S1. CNAG‐based visualization of single nucleotide variant (SNV) array data for the X chromosomes of the unbalanced X;19 translocation patient and the balanced carrier mother. Figure S2. Nexus‐based visualization of Affymetrix SNV array data in the proband (07‐966). [file NYAS-1561-0-s001.docx]

**Supplementary Material for**

**Escape from X-Chromosome Inactivation Enables Survival in a Male with an Unbalanced X;19 Translocation**

**Running Title:**

Escape from XCI in an X;19 Translocation

**Authors:**

Onur Emre Onat^1,2,*^, Tayfun Ozcelik^3,4,5^

1. Beykoz Institute of Life Sciences and Biotechnology, Bezmialem Vakıf University, İstanbul, Türkiye
2. Department of Molecular Biology, Bezmialem Vakıf University, İstanbul, Türkiye
3. Department of Molecular Biology and Genetics, Bilkent University, 06800 Ankara, Türkiye
4. Neuroscience Program, Graduate School of Engineering and Science, Bilkent University, 06800 Ankara, Türkiye.
5. Institute of Materials Science and Nanotechnology, National Nanotechnology Research Center, Bilkent University, 06800 Ankara, Türkiye.

*** Corresponding Author:**

E-mail: [onur.onat@bezmialem.edu.tr](mailto:onur.onat@bezmialem.edu.tr) (OEO)

**Number of Tables: 5**

**Number of Figures: 2**

**Table S1.** Genes located within the deleted subtelomeric interval at 19p13.3 (0–500 kb), extracted from Ensembl BioMart (version 0.7). The shaded region highlights the interval corresponding to the probable translocation breakpoint.

| **Cyto Band** | **Gene Start** | **Gene End** | **Gene Name** | **Description** |
| --- | --- | --- | --- | --- |
| 19p13.3 | 71973 | 72110 | hsa-mir-1302-2 | hsa-mir-1302-2 |
| 19p13.3 | 76163 | 77690 | FAM138E | Retina-specific protein F379 |
| 19p13.3 | 94659 | 96511 | AC008993.3 | |
| 19p13.3 | 104601 | 105256 | AC008977.1 | |
| 19p13.3 | 107146 | 111696 | OR4F17 | Olfactory receptor 4F17 |
| 19p13.3 | 197844 | 202173 | AC092299.1 | cDNA FLJ45445 |
| 19p13.3 | 198089 | 200744 | AC010507.1 | |
| 19p13.3 | 223158 | 223261 | U6 | U6 spliceosomal RNA |
| 19p13.3 | 223158 | 223260 | AC010507.3 | |
| 19p13.3 | 281048 | 291435 | PPAP2C | Lipid phosphate phosphohydrolase 2 |
| 19p13.3 | 305575 | 344791 | MIER2 | Mesoderm induction early response protein 2 |
| 19p13.3 | 362059 | 376009 | THEG | Testicular haploid expressed gene protein |
| 19p13.3 | 405443 | 409170 | FAM148C | Nuclear-localized factor 3 |
| 19p13.3 | 416587 | 460996 | SHC2 | SHC-transforming protein 2 |
| 19p13.3 | 453134 | 453239 | AC006124.1 | |
| 19p13.3 | 463344 | 474983 | ODF3L2 | Outer dense fiber protein 3-like protein 2 |
| 19p13.3 | 496454 | 505340 | MADCAM1 | Mucosal addressin cell adhesion molecule 1 |

**Table S2.** Genes flanking the translocation breakpoint on Xq11.1–11.2 (62.5–63.5 Mb), extracted from Ensembl BioMart (version 0.7). The shaded region denotes the interval most closely associated with the structural breakpoint.

| **Cyto**  **Band** | **Gene Start** | **Gene End** | **Gene Name** | **Description** |
| --- | --- | --- | --- | --- |
| Xq11.1 | 62519126 | 62519552 | RP13-34C21.1 |  |
| Xq11.1 | 62567107 | 62571218 | SPIN4 | Spindlin-4 |
| Xq11.1 | 62569525 | 62572057 | AL158203.1 |  |
| Xq11.1 | 62646439 | 62780929 | RP11-357C3.1 | hypothetical LOC92249, non-coding RNA |
| Xq11.1 | 62854847 | 63005413 | ARHGEF9 | Rho guanine nucleotide exchange factor 9 |
| Xq11.1 | 62890076 | 62891382 | AL391277.1 |  |
| Xq11.1 | 62986755 | 62987280 | AL451106.2 |  |
| Xq11.2 | 63005882 | 63005967 | hsa-mir-1468 | hsa-mir-1468 |
| Xq11.2 | 63264185 | 63264938 | AL356003.1 |  |
| Xq11.2 | 63364752 | 63366932 | AL355852.1 |  |
| Xq11.2 | 63404997 | 63425624 | FAM123B | Wilms tumor gene on the X chromosome |
| Xq11.2 | 63430571 | 63430866 | SRP_euk_arch | Eukaryotic signal recognition particle RNA |
| Xq11.2 | 63430571 | 63430868 | AL355852.3 |  |
| Xq11.2 | 63444076 | 63615333 | ASB12 | Ankyrin repeat and SOCS box protein 12 |
| Xq11.2 | 63487961 | 63615311 | MTMR8 | Myotubularin-related protein 8 |

**Table S3.** Primer sets and restriction enzymes used for allele-specific expression assays. This table lists the selected genes, genomic coordinates (GRCh38), primer sequences, amplicon sizes, restriction enzymes, recognition sites, and expected digestion products for the analyzed SNVs.

| **Gene** | **Chr** | **Forward & Reverse Primer** | **Size (bp)** | **Enzymes** | **Rec Site** | **1st Allele** | **2nd Allele** | |
| --- | --- | --- | --- | --- | --- | --- | --- | --- |
| NDUFS7 | 19p13.33 | CTTCCGGATCCTTGGTCTG  GTCATGGGCCACAGAGAACT | 216 | HpaII | C^CGG | C:4,31,107,51,23 | T:4,138,51,23 | |
| UBXD1 | 19p13.3 | GTTCAAGGCCGACATCAAGT  TTCGGCTTGAAGTTCCTTTC | 250 | Hpy188III | TC^NNGA | C:22,228 | T:- |  |
| DUS3L | 19p13.3 | CCCTCCCTAATCCAGGAGTC  CTCCTGCACCAGGTTCTGTC | 210 | DdeI | C^TNAG | A:25,152,35 | G:25,187 | |
| LASS4 | 19p13.2 | ATGTCAAGCGCAAGGATTTC  GAGGACCAGTCGGGTGTAGA | 239 | NlaIII | CATG^ | C:167,72 | T:120,47,72 | |
| DNMT1 | 19p13.2 | GCACAAACTGACCTGCTTCA  TGCCATTAACACCACCTTCA | 155 | BsmBI | CGTCTCN^ | A:- | G:135,20 | |
| MAST1 | 19p13.2 | GCAGATGGAGGAGAAGCTG  TGAGTAACGAAGGCCACCTC | 246 | HphI | ^NNNNNNNTCACC | A:139,107 | G:19,120,107 | |
| NDUFB7 | 19p13.1 | CCGGGTAGGAGCTAGGTGAC  CATCATCTCCTGCTGTGTGG | 197 | NlaIV | GGN^NCC | C:62,41,94 | G:103,94 | |
| MYO9B | 19p13.1 | CAGGCTGCCGTGTACCTC  CTTCTGCCTTCTGCTTCTCC | 160 | StuI | AGG^CCT | T:22,138 | G:- | |
| COPE | 19p13.11 | ATCTTCCAGGAGATGGCTGA  CTGTAGCACCAGCCTGTCAA | 300 | HpaII | C^CGG | C:139,161 | T:- | |
| FXYD5 | 19q12-q13.1 | CACCAGAGGACAGACGTTGA  ATGAGCTGCTTTGGTGCTCT | 244 | HinfI | G^ANTC | G:- | T:47,197 | |
| ECH1 | 19q13.1 | TACTGACCCGGCGACTGA  TTGAGCTGGACATGCAGAAC | 178 | AciI | G^CGG | A:- | C:81,97 | |
| ERCC2 | 19q13.3 | GCAGTACCAGCATGACACCA  TTGGCATGCAGGATTGAGTA | 177 | HinfI | G^ANTC | G:- | T:36,141 | |
| CARD8 | 19q13.32 | TCCTCCTTTCTCAGGTGCAG  ATTCTTGCTCTGCCGTGTCT | 163 | PspGI | ^CCWGG | G:53,37,63 | T:53,100 | |
| CD33 | 19q13.3 | CGGGAAGGAGCCATTATATC  CCTGTGGGTCAAGTCTGTCA | 249 | HpaII | C^CGG | A:- | G:20,229 | |
| RDH13 | 19q13.42 | CAGTTTGGCGTTAACCACCT  AGTCACACCAGAGCCTTGC | 249 | DdeI | C^TNAG | C:66,151,32 | T:66,183 | |

**Table S4.** Summary of semi-quantitative interpretation of the allele-specific expression assays shown in Figure 4. This table summarizes whether each locus was informative in the proband and indicates the overall interpretation of the corresponding restriction-digestion pattern.

| Gene | SNV | Proband informative? | Proband genotype | Densitometry summary in proband (07-966) | Interpretation | Confidence |
| --- | --- | --- | --- | --- | --- | --- |
| UBXD1 | rs1044510 | No | C/C | 228 bp dominant; alternate allele is minimal; no heterozygous interpretation possible | Non-informative in proband | Moderate |
| DUS3L | rs2436487 | No | A/A | Homozygous for reference allele | Non-informative in proband | High |
| LASS4 | rs1127912 | No | C/C | 120 bp band absent or minimal; no heterozygous interpretation possible | Non-informative in proband | Moderate |
| MYO9B | rs1545620 | No | T/T | Homozygous for reference allele | Non-informative in proband | High |
| FXYD5 | rs1688005 | No | T/T | Homozygous for alternate allele | Non-informative in proband | High |
| CD33 | rs2455069 | No | A/A | Homozygous for reference allele | Non-informative in proband | High |
| NDUFS7 | rs1142530 | Yes | C/T | Heterozygotes showed similar expression patterns and have weaker intensity when compared to homozygote mother | Compatible with biallelic expression / no convincing evidence for complete silencing | Moderate |
| NDUFB7 | rs9543 | Yes | C/G | Heterozygous family members showed broadly similar expression patterns, although band intensity differences limited strong quantitative interpretation. | Compatible with biallelic expression / escape | Low- Moderate |
| DNMT1 | rs2228611 | Yes | A/G | Proband and sister showed similar heterozygous expression patterns. Proband has slightly weaker intensity at the alternative allele when compared to mother. | Compatible with biallelic expression / escape | Moderate |
| COPE | rs2074797 | Yes | C/T | Heterozygotes showed variable expression patterns. When compared the alternate allele intensities, proband has higher expression than sister but lower than mother | Supports biallelic expression / escape | Moderate- High |
| ECH1 | rs9419 | Yes | A/C | Heterozygotes showed variable expression patterns. Proband has clearly weaker intensity at the alternate allele when compared to heterozygous mother. Substantial lane variability makes it difficult to interpret | Compatible with biallelic expression / no convincing evidence for complete silencing | Low |
| ERCC2 | rs238406 | Yes | G/T | Clearly proband shows similar intensities to other heterozygotes. Homozygous mother differs clearly from heterozygotes. | Supports biallelic expression / escape | High |
| CARD8 | rs3745718 | Yes | G/T | Weak heterozygous lane; mixed pattern consistent with both alleles, but overall signal is faint. Heterozygotes have weaker intensity when compared to homozygote mother | Compatible with biallelic expression / escape | Low |
| RDH13 | rs2305543 | Yes | C/T | Proband and sister showed similar heterozygous expression patterns. Although proband appeared to have weaker intensity for one allele than the homozygous mother, the overall pattern did not support complete silencing. | Supports biallelic expression / no convincing evidence for complete silencing | Moderate |

**Table S5.** Semi-quantitative densitometric summary of informative allele-specific expression assays shown in Figure 4. Band intensities were estimated from gel images using background-subtracted integrated density measurements and are reported in arbitrary units. Values should be interpreted as semi-quantitative. Closely spaced lower fragments were merged where reliable separation was not possible. Because some loci showed weak bands or limited resolution, these measurements were used only as supportive evidence for visual interpretation.

|  |  | 07-965 | | 07-966 | | 07-967 | | 07-968 | |
| --- | --- | --- | --- | --- | --- | --- | --- | --- | --- |
| **Gene (Variant)** | **Band (Allele)** | **Intensity** | **Percentage** | **Intensity** | **Percentage** | **Intensity** | **Percentage** | **Intensity** | **Percentage** |
| NDUFS7 (rs1142530) | 138 bp (T) | 10164 | 73,4% | 15273 | 77,1% | 21269 | 98,2% | 15854 | 78,5% |
|  | 107 bp (C) | 3676 | 26,6% | 4524 | 22,9% | 379 | 1,8% | 4349 | 21,5% |
| NDUFB7 (rs9543) | 103/94 bp (C) | 5770 | 82,3% | 14219 | 55,6% | 14450 | 51,3% | 14065 | 50,8% |
|  | 62 bp (G) | 1236 | 17,7% | 11340 | 44,4% | 13688 | 48,7% | 13641 | 49,2% |
| DNMT1 (rs2228611) | 155 bp (A) | 3509 | 80,8% | 8231 | 78,5% | 0 | 0,0% | 0 | 0,0% |
|  | 135 bp (G) | 835 | 19,2% | 2259 | 21,5% | 11192 | 100% | 8338 | 100,0% |
| COPE (rs2074797) | 300 bp (T) | 8230 | 58,0% | 11249 | 51,7% | 12527 | 50,1% | 0 | 0,0% |
|  | 161/139 bp (C) | 5959 | 42,0% | 10511 | 48,3% | 12462 | 49,9% | 32149 | 100,0% |
| ECH1 (rs9419) | 178 bp (C) | 13024 | 100,0% | 13118 | 88,4% | 13651 | 78,7% | 2968 | 17,8% |
|  | 97/81 bp (A) | 0 | 0,0% | 1714 | 11,6% | 3698 | 21,3% | 13722 | 82,2% |
| ERCC2 (rs238406) | 177 bp (G) | 10096 | 64,5% | 12966 | 68,6% | 20428 | 100,0% | 15528 | 65,0% |
|  | 141 bp (T) | 5546 | 35,5% | 5930 | 31,4% | 0 | 0,0% | 8355 | 35,0% |
| CARD8 (rs3745718) | 100 bp (G) | 1861 | 53,9% | 1999 | 59,3% | 13510 | 71,6% | 0 | 0,0% |
|  | 67 bp (T) | 1219 | 35,3% | 1010 | 30,0% | 0 | 0,0% | 6236 | 80,6% |
|  | 53 bp (G) | 371 | 10,8% | 360 | 10,7% | 5351 | 28,4% | 1501 | 19,4% |
| RDH13 (rs2305543) | 183 bp (T) | 10969 | 72,1% | 13165 | 84,3% | 0 | 0,0% | 0 | 0,0% |
|  | 151 bp (C) | 4240 | 27,9% | 2447 | 15,7% | 22949 | 100,0% | 22344 | 100,0% |


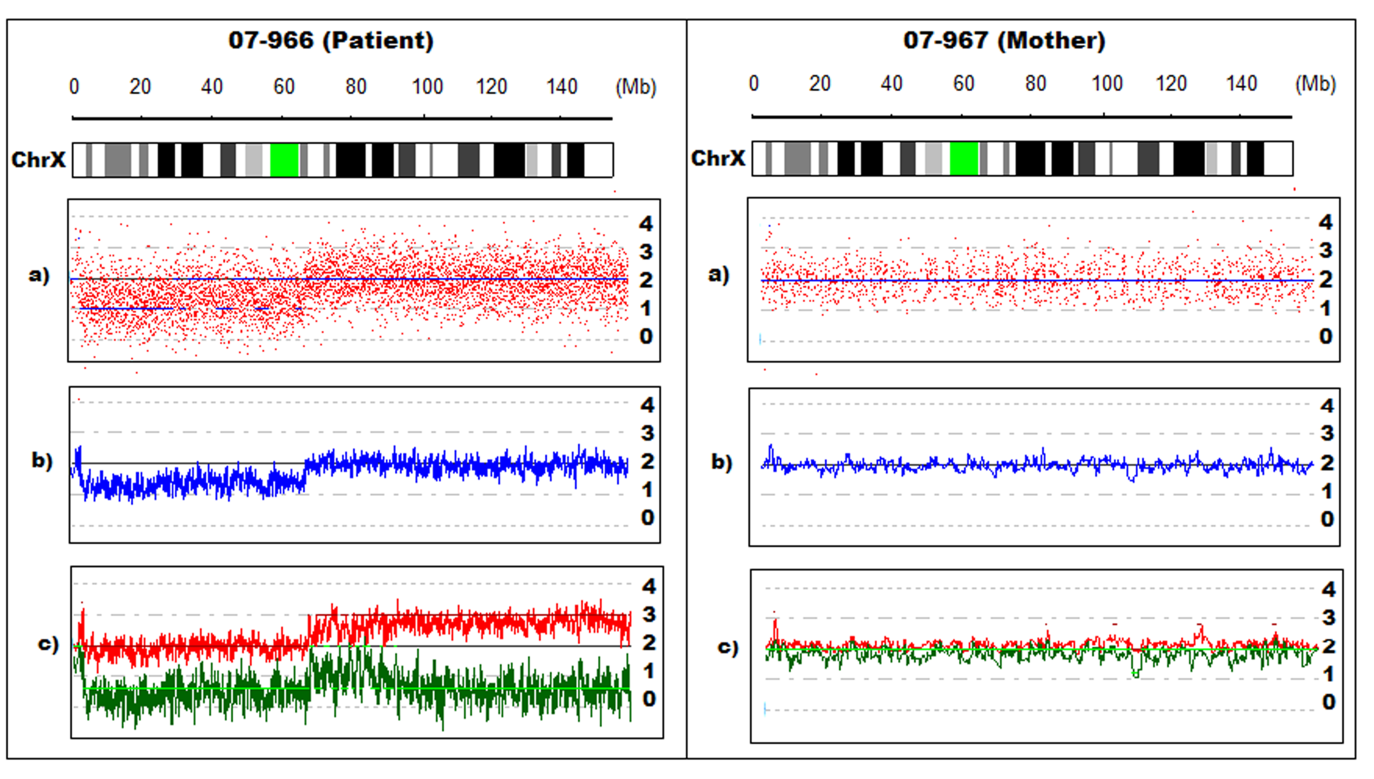


**Figure S1.** CNAG-based visualization of SNV array data for the X chromosomes of the unbalanced X;19 translocation patient and the balanced carrier mother. **A.** Copy-number state profile of the X chromosome generated using Copy Number Analyzer for GeneChip (CNAG). **B.** CNAG copy-number display using the Hidden Markov Model (HMM) color gradient. **C.** Allele-based analysis showing B-allele frequencies across the genome. The left panels show the proband (07-966), and the right panels show the mother (07-967).


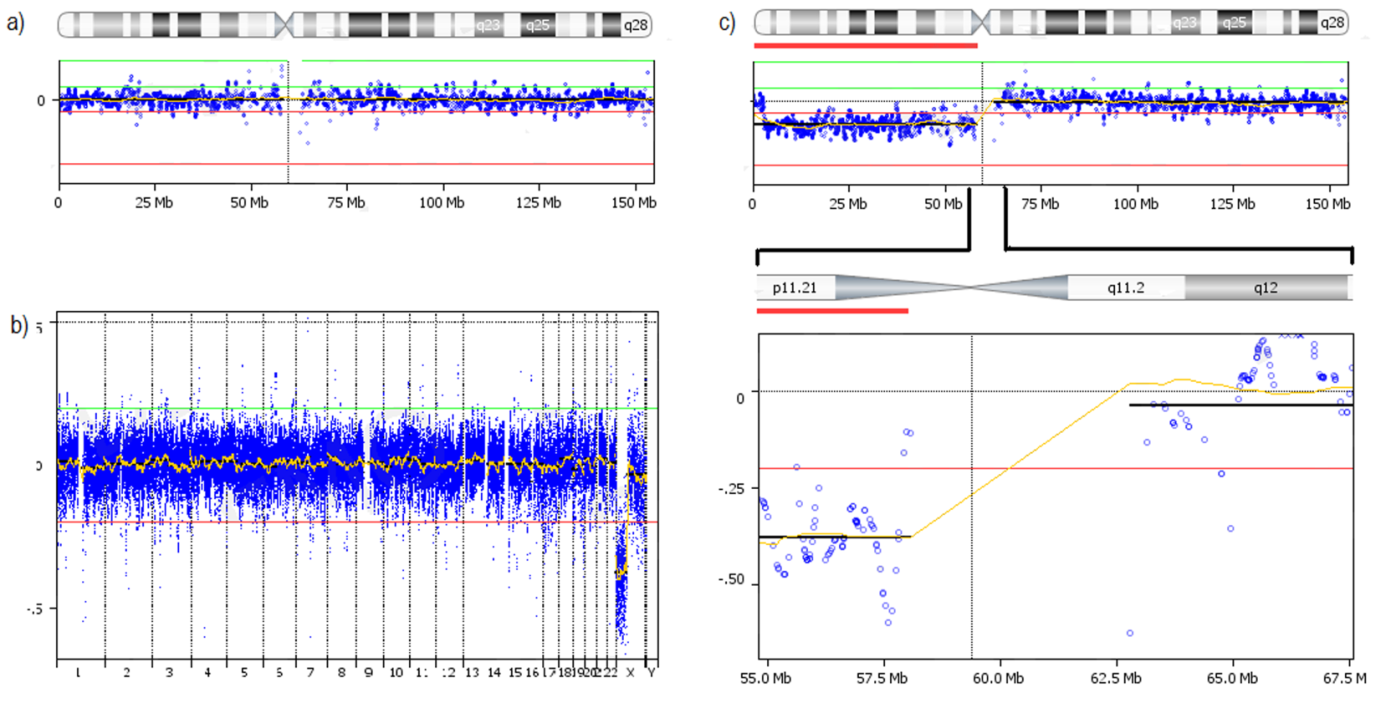


**Figure S2.** Nexus-based visualization of Affymetrix SNV array data in the proband (07-966). **A.** Copy-number state profile of the X chromosome. **B.** Genome-wide copy-number profile. **C.** Expanded view of X-chromosome copy-number states showing the Xq11.1→Xqter duplication.
